# A Case of Posterior Reversible Encephalopathy Due to Takotsubo Cardiomyopathy Triggered by Aerophobia

**DOI:** 10.7759/cureus.40678

**Published:** 2023-06-20

**Authors:** Güldeniz Çetin, Esra Nur Sancar, Serkan Demir, Özdem Ertürk Çetin, Sevki Sahin

**Affiliations:** 1 Neurology, Dr. İlhan Varank Sancaktepe Research and Training Hospital, Istanbul, TUR; 2 Neurology, Sancaktepe Sehit Prof. Dr. Ilhan Varank Training and Research Hospital, İstanbul, TUR; 3 Neurology, University of Health Sciences, Sancaktepe Şehit Prof. Dr. İlhan Varank Training and Research Hospital, İstanbul, TUR

**Keywords:** loss of conciousness, unexplained syncope, heart failure, posterior reversible encephalopathy syndrome (pres), tako-tsubo cardiomyopathy (ttc)

## Abstract

A 37-year-old woman, previously known to have severe airplane phobia, develops panic disorder-like symptoms in the second hour of the flight. After a while, panic disorder was accompanied by chest pain and syncope. An ECG showed no abnormalities. Transthoracic echocardiogram demonstrated decreased left ventricular ejection fraction (EF: 30), large apical akinesis, and characteristic regional wall motion abnormalities involving the middle and apical segments of the left ventricle. Findings were consistent with Takotsubo cardiomyopathy. But in the emergency department, Brain Diffusion MRI showed cortical and subcortical vasogenic edema in the posterior regions, predominantly on the left, bilaterally, compatible with posterior reversible encephalopathy. This case highlights the Takotsubo cardiomyopathy-related posterior reversible encephalopathy syndrome (PRES) syndrome and managing the disease.

## Introduction

Although Takotsubo is cardiomyopathy that mimics myocardial infarction, it differs in its pathophysiology in that the microvascular spasm that occurs after emotional stress leads to a reversible clinical scenario. This condition may rarely affect the cerebrovascular circulation, simultaneously leading to posterior reversible encephalopathy syndrome (PRES). Aerophobia is an extreme fear of flying by airplane. People with aerophobia may fear different aspects of flight, such as taking off, landing, or being trapped in an airplane. This case report aimed to demonstrate the Takotsubo cardiomyopathy (TC) associated with PRES in a patient with aerophobia.

## Case presentation

**Figure 1:**
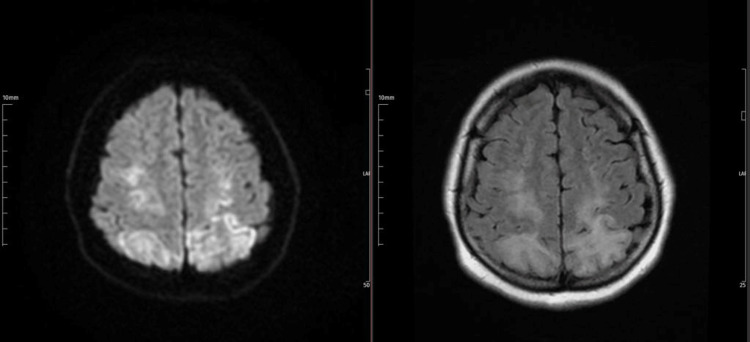
Axial diffusion-weighted magnetic resonance imaging showed prominent bilateral hyperintensity on the posterior regions of the brain's left side consistent with posterior reversible encephalopathy.

**Figure 2:**
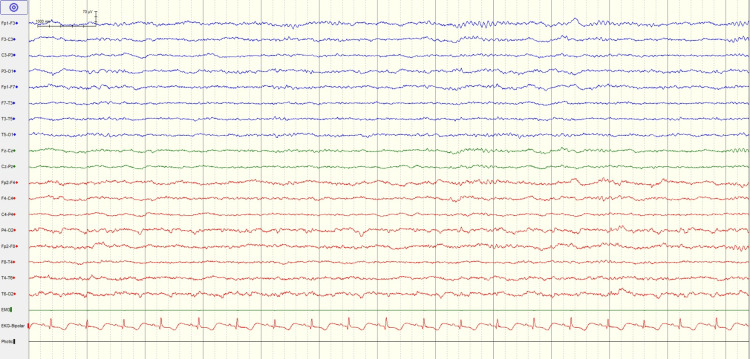
Epileptiform activity was not observed on the EEG

A 37-year-old woman with previously known severe airplane phobia developed panic-like symptoms during the second hour of the flight. After a while, the panic disorder was accompanied by chest pain and syncope. She was rushed to the emergency room in a disoriented state, exhibiting notable limitations in orientation and cooperation and diffusing myoclonic spasms throughout her body. Afterward, the patient began to experience agitation. ECG and troponin follow-ups are performed to exclude the considering acute coronary syndrome in the foreground. The initial troponin level was 489 ng/L (normal range: 0-14 ng/L) but then normalized in four hours, which helped rule out myocardial infarction. Echocardiography shows decreased left ventricular ejection fraction, large akinesia of the apex, and characteristic regional wall motion abnormalities of the middle and apex of the left ventricle. Brain diffusion magnetic resonance imaging (MRI) is performed in the emergency department. It is consistent with cortical and subcortical vasogenic edema in the posterior regions, predominantly on the left side and bilateral, compatible with posterior reversible encephalopathy (PRES) (Figure 1). Brain computed angiography was normal. Epileptiform activity was not observed on the EEG (Figure 2). Clinical improvement was achieved within six weeks, confirming the diagnosis of Takotsubo and PRES.

## Discussion

Takotsubo Cardiomyopathy (TC), initially reported in 1990 [1], refers to a condition where there is temporary acute heart failure and left ventricular enlargement as a response to intense emotional or physical stress [2]. As per the Revised Mayo Clinic Criteria, the diagnosis of Takotsubo Cardiomyopathy necessitates the presence of four specific conditions: temporary dyskinesis affecting the midsegments of the left ventricle (with or without the involvement of the apex), regional wall motion abnormalities that extend beyond the distribution of a single epicardial blood vessel and are triggered by a stressful event; the absence of obstructive coronary disease or angiographic evidence of sudden plaque rupture; new electrocardiogram (ECG) abnormalities such as ST-segment elevation and/or T-wave inversion, or a moderate increase in cardiac troponin levels and the exclusion of pheochromocytoma and myocarditis [3].

Patients typically present with chest pain, dyspnea, dizziness, generalized weakness, and syncope. Physical examination findings resembling acute systolic heart failure include jugular vein distension, an S3 gallop, tachycardia (rapid heart rate), hypotension (low blood pressure), and reduced pulse pressure. Additionally, there is a possibility of developing a systolic ejection murmur caused by obstruction of the left ventricular outflow tract and mitral regurgitation due to ventricular ballooning. Most patients display elevated levels of troponin T, I or creatine kinase-MB (CK-MB), ST elevation, T-wave inversion on electrocardiograms (ECGs), and increased brain natriuretic peptide (BNP) [4].

The exact pathophysiology of Takotsubo cardiomyopathy remains unclear; however, several pathological mechanisms have been proposed. The correlation between stressful triggers and TC indicates the significant involvement of the adrenergic system in its pathophysiology. It is believed that catecholamines, including epinephrine and norepinephrine, released during times of stress, play a role in the development of apical ballooning, potentially leading to myocardial toxicity and/or disruption of the cardiac microvasculature [4-7]. However, a recent study has indicated that catecholamine discharge is a shared consequence of PRES and Takotsubo, suggesting an overlap between PRES and Takotsubo and an unclear cause-effect relationship [8].

Increased serum and cardiac nerve catecholamines in the acute period are believed to contribute to vasospasm in both epicardial and cardiac vessels. Vasospasm increases cardiac workload, impairs tissue perfusion, and leads to symptoms similar to ischemia [9]. Vascular dysfunction is thought to be a contributing factor in this mechanism [10].

Although the treatment of Takotsubo cardiomyopathy remains uncertain, patients are usually monitored in cardiology wards for 21 days to assess left ventricular systolic function. Mild to moderate cases are managed symptomatically, while severe cases may require mechanical ventricular support [4].

Despite the generally good prognosis associated with TC, complications carry a higher mortality risk. Currently, no standardized guidelines exist for the diagnostic and management approach to TC. Conservative management is generally effective [11]. Initially, it is important to ensure the hemodynamic stability of patients. If pulmonary congestion is present, diuretics and vasodilators (such as nitroglycerin, nitroprusside, or nesiritide), as well as medications like beta-blockers, angiotensin-converting enzyme (ACE) inhibitors, and angiotensin II receptor blockers (ARBs), are used to lower the heart rate. In refractory cases, vasopressors and left ventricular assist devices may be necessary [4]. The treatment of TC-related arrhythmias with a cardiac pacemaker or implantable cardioverter-defibrillator (ICD) remains controversial due to the reversible nature of the condition [11].

When admitting a patient with Takotsubo cardiomyopathy, clinicians should know the high risk (7%) of cerebrovascular events in the first 30 days [6]. Atrial fibrillation may develop in patients with large cardiac hypokinetic areas [4].

Posterior reversible encephalopathy syndrome (PRES) is a cerebrovascular event characterized by acute onset of altered consciousness, headache, visual loss (visual hallucinations, cortical blindness, hemianopia, diplopia), and sometimes epileptic seizures [12]. Risk factors include sepsis, preeclampsia, liver and kidney diseases, immunosuppressant and nephrotoxic drug use, and autoimmune diseases [13]. Uncontrolled hypertension is the most common factor among these risk factors [14].

Increased hydrostatic pressure disrupts the blood-brain barrier, leading to extravasation of intravascular fluid into the brain tissue and brain tissue edema. Parietooccipital regions are commonly affected, less commonly temporal and frontal lobes, brainstem, and deep white matter. The posterior system is more affected than the anterior system due to its limited adaptive mechanisms to regulate the blood-brain barrier [15].

The clinical and radiological features give PRES its name. The gold standard for diagnosis is to demonstrate vasogenic edema on non-contrast MRI as T2 hyperintense, predominantly in the parieto-occipital lobes [14]. Cranial MR angiography (MRA), which is typically normal in PRES, helps to exclude CNS vasculitis, and normal magnetic resonance venography (MRV) helps to exclude possible sagittal sinus thrombosis [16]. There is no specific pattern in EEG imaging [17].

Treatment is usually directed toward the underlying cause. In the presence of hypertension, a standard protocol has not been established. It is recommended to initiate antihypertensive treatment when blood pressure reaches 160/100 mm Hg. Sudden blood pressure lowering may increase the risk of cerebral hypoperfusion and ischemia. Therefore, blood pressure should not be reduced by more than 10-20 mm Hg every 10-20 minutes [18]''.

## Conclusions

Patients experiencing chest pain and dyspnea following physically or mentally stressful triggers; should exercise caution for TC, a condition that imitates acute myocardial infarction. Close monitoring is essential to ensure early diagnosis and promptly address cerebrovascular events' potential occurrence. Risk factors should be rapidly identified, and the underlying etiology should be treated. Our case is one of the rare cases in which TC is associated with PRES. The pathogenesis is believed to involve adrenergic discharge triggered by aerophobia. When recent cases are examined, the cause-effect relationship between Takotsubo and PRES is not clearly established. Therefore, more clinical research is needed.
